# Machine learning applied on chest x-ray can aid in the diagnosis of COVID-19: a first experience from Lombardy, Italy

**DOI:** 10.1186/s41747-020-00203-z

**Published:** 2021-02-02

**Authors:** Isabella Castiglioni, Davide Ippolito, Matteo Interlenghi, Caterina Beatrice Monti, Christian Salvatore, Simone Schiaffino, Annalisa Polidori, Davide Gandola, Cristina Messa, Francesco Sardanelli

**Affiliations:** 1 Department of Physics, Università degli Studi di Milano-Bicocca, Piazza della Scienza 3, 20126 Milan, Italy; 2 Institute of Biomedical Imaging and Physiology, National Research Council, 20090 Segrate, Milan, Italy; 3 Department of Radiology, San Gerardo Hospital, Via Pergolesi 33, 20900 Monza, Italy; 4 Department of Biomedical Sciences for Health, Università degli Studi di Milano, Via Mangiagalli 31, 20133 Milan, Italy; 5 Scuola Universitaria Superiore IUSS Pavia, Piazza della Vittoria 15, 27100 Pavia, Italy; 6 DeepTrace Technologies S.R.L., Via Conservatorio 17, 20122 Milan, Italy; 7 Department of Radiology, IRCCS Policlinico San Donato, Via Morandi 30, 20097 San Donato Milanese, Milan, Italy; 8 School of Medicine and Surgery, University of Milano-Bicocca, Piazza dell’Ateneo Nuovo 1, 20126 Milan, Italy; 9 Fondazione Tecnomed, Università degli Studi di Milano-Bicocca, Palazzina Ciclotrone, Via Pergolesi 33, 20900 Monza, Italy

**Keywords:** Artificial intelligence, COVID-19, Neural networks (computer), Sensitivity and specificity, X-rays

## Abstract

**Background:**

We aimed to train and test a deep learning classifier to support the diagnosis of coronavirus disease 2019 (COVID-19) using chest x-ray (CXR) on a cohort of subjects from two hospitals in Lombardy, Italy.

**Methods:**

We used for training and validation an ensemble of ten convolutional neural networks (CNNs) with mainly bedside CXRs of 250 COVID-19 and 250 non-COVID-19 subjects from two hospitals (Centres 1 and 2). We then tested such system on bedside CXRs of an independent group of 110 patients (74 COVID-19, 36 non-COVID-19) from one of the two hospitals. A retrospective reading was performed by two radiologists in the absence of any clinical information, with the aim to differentiate COVID-19 from non-COVID-19 patients. Real-time polymerase chain reaction served as the reference standard.

**Results:**

At 10-fold cross-validation, our deep learning model classified COVID-19 and non-COVID-19 patients with 0.78 sensitivity (95% confidence interval [CI] 0.74–0.81), 0.82 specificity (95% CI 0.78–0.85), and 0.89 area under the curve (AUC) (95% CI 0.86–0.91). For the independent dataset, deep learning showed 0.80 sensitivity (95% CI 0.72–0.86) (59/74), 0.81 specificity (29/36) (95% CI 0.73–0.87), and 0.81 AUC (95% CI 0.73–0.87). Radiologists’ reading obtained 0.63 sensitivity (95% CI 0.52–0.74) and 0.78 specificity (95% CI 0.61–0.90) in Centre 1 and 0.64 sensitivity (95% CI 0.52–0.74) and 0.86 specificity (95% CI 0.71–0.95) in Centre 2.

**Conclusions:**

This preliminary experience based on ten CNNs trained on a limited training dataset shows an interesting potential of deep learning for COVID-19 diagnosis. Such tool is in training with new CXRs to further increase its performance.

## Key points

- A deep learning classifier was applied to chest x-rays of suspected COVID-19 patients.
- This method provided a balanced diagnostic performance with 0.80 sensitivity and 0.81 specificity.
- Training on larger multi-institutional datasets may allow such performance to increase.

## Background

According to the John Hopkins Coronavirus Resource Centre [1], as of September 2020, the severe acute respiratory syndrome coronavirus 2 (SARS-CoV-2) infected almost 30 million individuals with more than 900,000 deaths worldwide.

In this pandemic, clinicians are requesting fast diagnostic tools for SARS-CoV-2 infection and coronavirus disease 2019 (COVID-19) characterised by a good balance between sensitivity and specificity, leading to acceptable predictive values in a context of a variable prevalence. Of note, any tool to be applied for this aim should have a good cost-benefit ratio for the healthcare service.

The clinical standard for detecting coronavirus infections is reverse transcriptase polymerase chain reaction (RT-PCR) [2], even though this test can give a false negative result at an early stage of the disease and the time needed to get its result is highly variable. At any rate, considering the most relevant clinical evolution leading to pneumonia, chest imaging study is routinely performed in suspected or confirmed COVID-19 cases, as suggested by the World Health Organization guidelines [3].

When a patient presents with symptoms attributable to COVID-19, like fever, cough, or dyspnoea, chest x-ray (CXR) is usually the first imaging test performed, because it is cheaper and easier to do [4]. Furthermore, CXR can also be acquired with portable instrumentation in isolated rooms in emergency departments or at the patient’s bedside in every other department, which would considerably ease the required sanitisation process [5]. CXR images have a high spatial resolution, but they are planar images, not allowing three-dimensional slicing as all structures visualised at CXR are displayed on a single plane. Anyway, even for CXR, the most common reported abnormal finding is ground-glass opacities, with portions of the lungs appearing as a “hazy” shade of grey instead of being black with fine white lung markings for blood vessels [6]. As ground-glass opacities are usually the first radiological sign of COVID-19, it could be hypothesised to be able to improve the early diagnosis of COVID-19 by means of a smarter reading of CXRs.

Machine learning is emerging as a unique powerful method to improve the diagnosis and prognosis of several multifactorial diseases, including pneumonia. In 2018, a worldwide competition on the Kaggle portal (www.kaggle.com) was launched by the Radiological Society of North America on the complex task of automatically screening pneumonia (viral and bacterial) [7] *versus* non-pneumonia patients on CXRs, where leader groups obtained excellent results training their different machine learning systems on CXRs [8]. More recently, a Chinese research team proved the potential of machine learning in supporting the diagnosis of COVID-19 in the Chinese population suspected for COVID-19 when trained on CT images, showing excellent results, with sensitivity and specificity higher than 90% [9]. However, their machine learning CT-based model may not be implemented in an emergency context as for the SARS-CoV-2 pandemic. Concerning the use of machine learning on CXR images for COVID-19 detection, a number of studies reached satisfactory diagnostic accuracy by employing convolutional neural networks or other deep learning methods [10–15].

Thus, the aim of our study was to test a deep learning classifier applied to CXRs in the SARS-CoV-2 emergency setting also considering the radiologists’ reading performance. Our purpose was to develop a tool able to support the diagnosis of COVID-19, offering a second opinion to clinical radiologists worldwide.

## Methods

### Ethical approval

The local Ethics Committee (Ethics Committee of IRCCS San Raffaele) approved this retrospective study on 8 April 2020, and informed consent was waived due to the retrospective nature of the study. In our retrospective study, we used a case-control design based on non-consecutive patients and an artificially enriched positive class (COVID-19).

### Training and validation set

The training and validation set was composed of CXRs from (1) the Hospital San Gerardo, Monza, Italy (Centre 1) and (2) the IRCCS Policlinico San Donato (Centre 2).

For Centre 1, non-consecutive patients suspected for COVID-19 admitted from 1 March to 13 March 2020 (*n* = 270, 135 COVID-19 and 135 non-COVID-19) were considered. Clinical suspicion of COVID-19 was defined upon arrival at the emergency room and based on the referring physician judgement for patients admitted at the emergency department, taking into consideration onset of symptoms (the main fever, cough and dyspnoea) and blood tests (white blood cell count, red blood cell count, C-reactive protein level). All these patients suspect for pneumonia underwent digital CXR in anteroposterior projection at bedside as well as RT-PCR assays using commercial kits (ribonucleic acid was extracted from collected samples). The classification of positive or negative COVID-19 cases was based on the detection or non-detection of the pathogen: the number of cases of negative RT-PCR followed by one or more further negative swabs was 135. The CXR images from Centre 1 were obtained using two different imaging systems: WDR Mobile Diagnost (Philips, Amsterdam, The Netherlands), and DX-D100 (AGFA, Mortsel, Belgium).

Due to the higher prevalence of COVID-19 patients with respect to non-COVID-19 in the considered period, we were forced to “artificially” enrich the dataset in order to balance the number of patients’ images in the two classes because a balanced set of classes is recommended to properly train a deep learning classifier [16]. In other words, in training the algorithm, the sampling rate of the two classes of images (COVID-19 and non-COVID-19) was not equal to their actual prevalence, but the number of samples (images) of the class with lower prevalence (non-COVID-19) was chosen a posteriori to match the number of COVID-19 images in order to obtain a final dataset composed equally of patients’ images of the two classes.

For Centre 2, we included in the training and validation dataset of digital CXRs of consecutive patients suspected for COVID-19 according to the same criteria described above, admitted to the IRCCS Policlinico San Donato (Centre 2) from 25 February 25 to 16 March 2020 and subsequently confirmed to be COVID-19 positive by RT-PCR (*n* = 115). Out of these CXRs, 87 were anteroposterior projections performed at bedside and 28 posteroanterior projections acquired in upright position. This set of data was further enriched with CXRs of non-consecutive sex- and age-matched patients admitted to Centre 2 approximately in the same time interval of the previous year (*n* = 115, 15 February to 16 March 2019), who underwent CXR for pneumonia symptoms, without any mention of lung abnormalities in the radiological report. Of these CXRs, 16 were anteroposterior projections performed at bedside and 99 were posteroanterior projections acquired in upright position. No matching for severity was performed. The CXR images of Centre 2 were obtained using two different imaging systems: digital GM85 (Samsung, Seoul, South Korea) and digital FDR Go PLUS (Fujifilm, Tokyo, Japan).

### Independent testing set

We then retrospectively considered consecutive patients suspected of COVID-19 admitted to the Hospital San Gerardo, Monza, Italy (Centre 1) from 14 March to 19 March 2020, thus temporally separated from the training and validation set coming from the same centre. Clinical suspicion of COVID-19, digital bedside CXRs and specific RT-PCR assays were performed as previously described (*n* = 110), 74 of them resulted to be COVID-19 and 36 non-confirmed at RT-PCR assay.

**Fig. 1:**
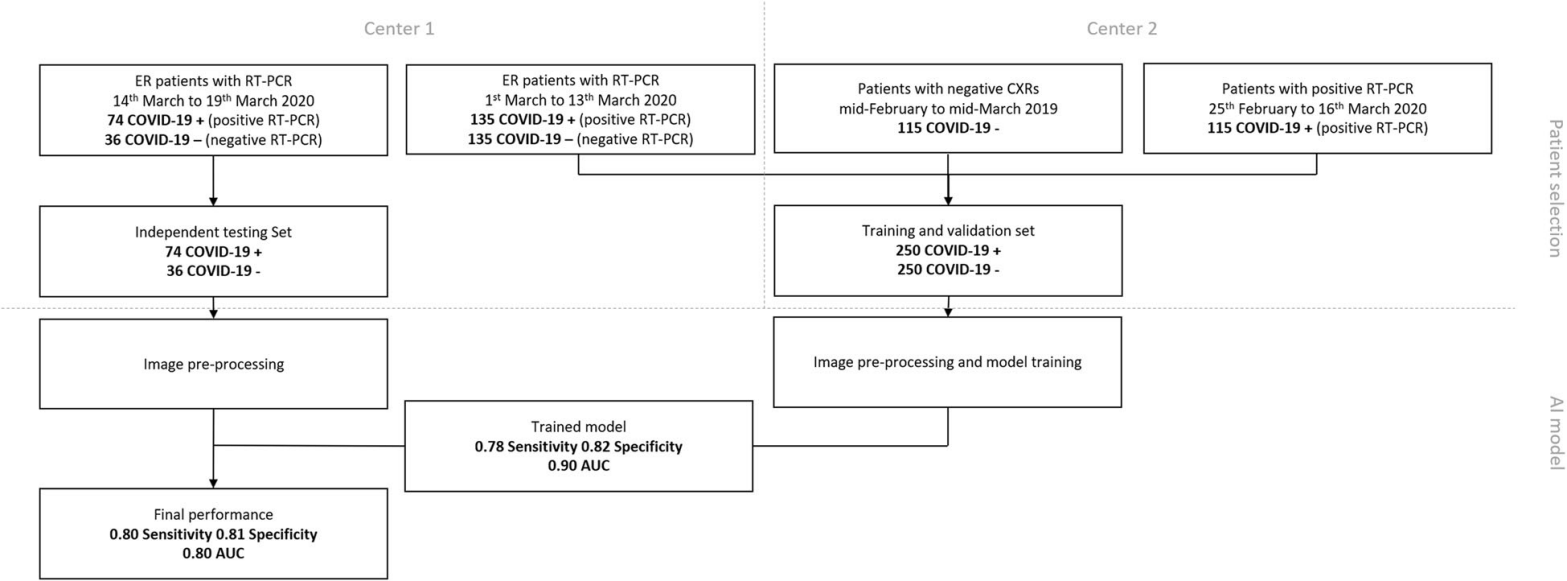
Flow diagram for patient selection. *CXR* Chest x-ray, *RT-PCR* Reverse transcriptase polymerase chain reaction, *ER* Emergency room, *AUC* Area under the curve

A flow diagram describing patient selection is depicted in Fig. 1, while Table 1 summarises included patients’ provenience and COVID-19 positivity or negativity.

**Table 1:** Provenience and characteristics of the included chest x-ray exams

	Timeframe	COVID-19	Negative	Total
	**Classifier training + validation (cross-validation)**			
**Centre 1**	1 March to 13 March 2020	135	135	270
**Centre 2**	25 February to 16 March 2020	115		230
	Mid-February to mid-March 2019		115	
**Total training + validation**		**250**	**250**	**500**
	**Classifier and human independent testing**			
**Centre 1**	14 March to 19 March 2020	74	36	110
**Total testing**		**74**	**36**	**110**
**Total training + validation + testing**		**324**	**276**	**610**

### Image analysis by deep learning

Starting from the included CXR images (Fig. 3), we tuned, trained, validated and tested a deep learning classifier in the binary classification task of interest (COVID-19 *versus* non-COVID-19). For tuning, training and validation, we considered (1) all patients with positive RT-PCR from both centres (*n* = 250) as COVID-19 and (2) all patients with negative RT-PCR from Centre 1 and those admitted to Centre 2 in the same period of the previous year with a CXR reported as negative (*n* = 250) as non-COVID-19 (see Table 1). For testing the deep learning classifier, we considered an “independent testing set” of 110 CXRs obtained from Centre 1, and temporally separated from the abovementioned set of 250 *versus* 250 CXRs used for the tuning, training and validation of the deep learning classifier (see the “Methods” section and the “Independent testing set” section for further details).

For these purposes, we used the TRACE4© software platform (http://www.deeptracetech.com/files/TechnicalSheet__TRACE4.pdf, DeepTrace Technologies, Italy), which allows (i) tuning, training, validation, and testing ensembles of a variety of convolutional neural networks (CNNs) with different architectures and (ii) processing medical images to easy match such CNNs’ input constraints (*e.g*., image size).

Among the available CNNs, we choose the ResNet-50 architecture [17], a CNN with an extremely deep architecture, composed of 50 layers of neurons able to learn a rich feature representation of the input image classes during training (*e.g.*, more than a million images from the ImageNet database, ImageNet. http://www.image-net.org). ResNet-50 uses these feature representations to classify new images as belonging to one of the input classes’ layers. ResNet-50 first preprocesses the input image by one convolutional layer (7 × 7) and one max pooling layer (3 × 3). Then, the preprocessed image is input to 4 blocks of layers of similar structure, including a different number of 3 × 3 convolutional filters providing different features’ map size. Each block filters the preprocessed image and sends forward the filtered image to the next block of convolutional filters. Moreover, some block skips the preprocessed image to the next block. This operation is called “skip connection” and represents one hallmark of ResNet architectures solving the vanishing gradient problem that implies that classifier performance gets saturated rapidly in extremely deep architectures.

The last layers of ResNet50 consist into one average pooling layer and one fully connected layer followed by a support vector machine (SVM) classifier layer.

**Fig. 2:**
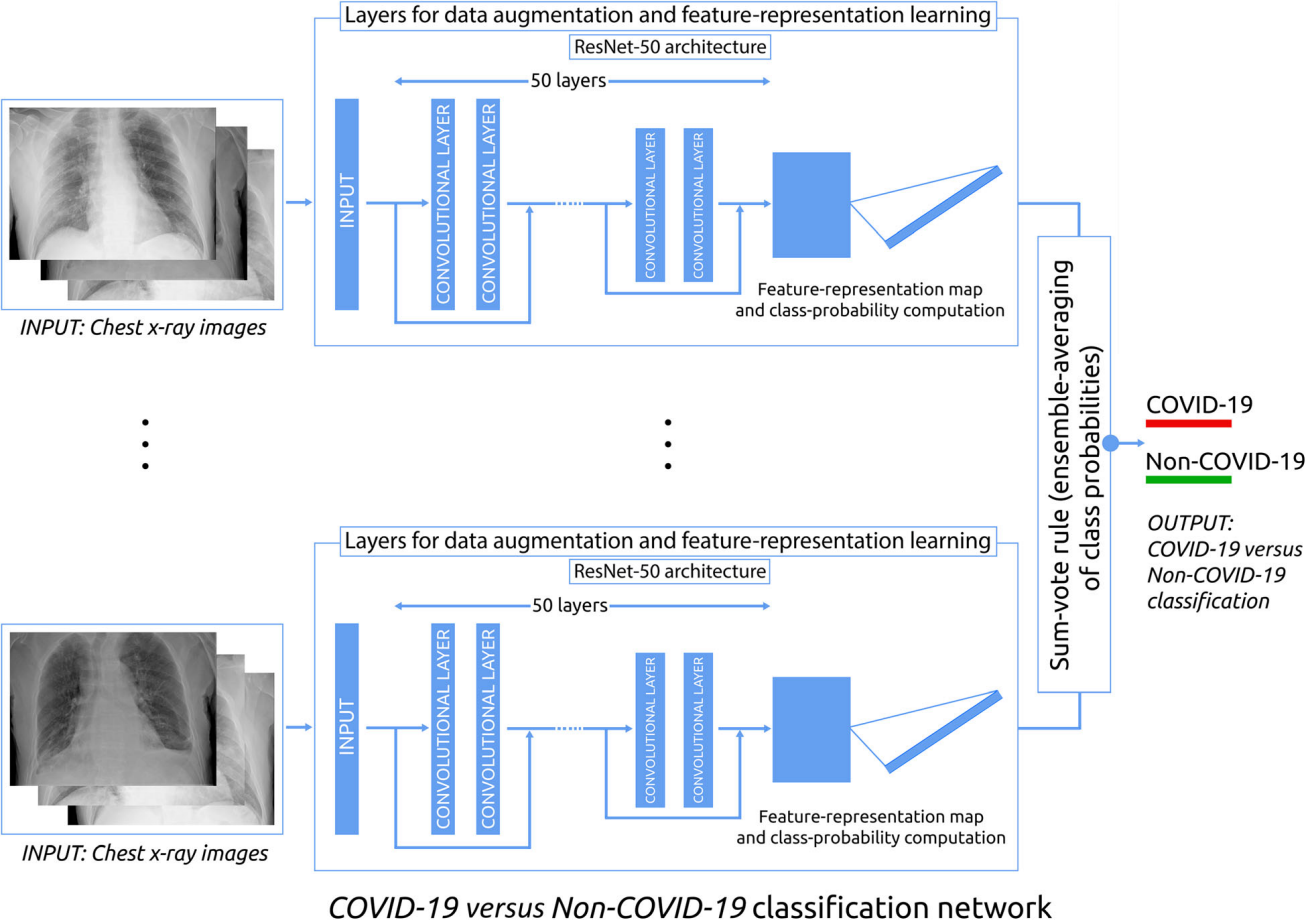
Schematic drawing of the artificial intelligence network architecture for the classification of COVID-19 *versus* non-COVID-19 patients through chest-x-ray imaging *COVID-19* Coronaviris disease 2019

A schematic drawing for the network architecture is presented in Fig. 2.

TRACE4 allows medical images fed directly into the CNNs following an automatic up- or downsampling depending on the different original image size. In this study, this purpose was obtained by a downsampling of approximately 1/5 in order for all CXR images to match the ResNet-50 input image size (224 by 224). No specific lung pattern was selected, and no annotations or segmentation were performed by radiologists on images.

In order to increase CXR diversity among different training phases (epochs), we provided data-augmentation and image-manipulation techniques that were applied to the 500 (250 + 250) CXR training image set, as follows: (1) 500 reflections in the top-bottom direction and 500 reflections in the left-right directions applied randomly (50% probability); (2) 500 planar rotations, with rotation angle picked randomly from a continuous uniform distribution from -5° to +5°; (3) 500 horizontal and 500 vertical shears, with shear angle picked randomly from a continuous uniform distribution from -0.05° to +0.05°.

TRACE4 allowed a fine-tuning of ResNet-50 architecture to specialise its two last layers to our binary classification task (COVID-19 *versus* non-COVID-19). Specifically, TRACE4 optimised during training the maximum number of epochs to 30, with an optimal mini-batch size of 8 (the samples of the training set were randomised before each epoch in order to avoid issues related to the choice of samples to include in the mini-batches—*e.g*., always discarding the same samples). An Adam optimisation was used for stochastic gradient computation [18]. TRACE4 did not optimise the learning rate that was maintained of 1e−4 constant throughout the whole training.

A 10-fold cross-validation method was chosen for training and validation. The division ratio between the training set and the validation set was 9:1. For each fold of the cross-validation, the ResNet-50 was trained on the training set and used to classify the validation set. The ResNet-50 was then used to obtain the classification performance on both the training and the validation sets. This resulted in 10 different ResNet-50-derived classification models and in a set of 10 classification performances (one for each fold). The final training and validation performance were calculated as the mean of the performances obtained on each of the 10 ResNet50.

Independent testing was performed using 10 ResNet-50 classifiers trained and validated as above in an ensemble strategy. Images of the independent testing set were classified using the 10 ResNet-50 classifiers, thus obtaining 10 classification outputs and 10 class-membership probabilities for each image (one for each ensemble classifier). The final classification for each image was calculated by the mean probabilities assigned by the 10 classifiers. Testing performance was then calculated over the entire independent testing set.

The performance obtained by such deep learning classifier for both cross-validation and independent testing were computed in terms of accuracy, sensitivity, specificity, area under the curve (AUC) at receiver operating characteristics analysis, positive likelihood ratio (LR+), and negative likelihood ratio (LR-), with their corresponding 95% confidence interval (CI).

### Image analysis by radiologists

A retrospective reading of CXRs was performed by staff radiologists at both hospitals. They were one radiologist with 15 years of experience in chest imaging at Centre 1 (Reader 1) and a general radiologist with 6 years of experience at Centre 2 (Reader 2). They were asked to standardise the reading without any information on medical history, clinical and biologic data, with the aim to differentiate COVID-19 patients from non-COVID-19 patients. Both readers assessed the independent testing cases consisting into 110 bedside CXRs of patients suspected to be COVID-19 infected, all from the emergency department of Centre 1 from 16 March to 19 March 2020, 74 of them finally resulting positive for COVID-19 at RT-PCR and 36 negative for COVID-19 at RT-PCR (see Table 1). Readers were asked to provide an overall judgement on whether each CXR image was positive or negative.

Results of human reading performance (sensitivity, specificity, LR+, and LR-) were computed and presented as ratios with their 95% CI.

## Results

### Patient population

Our training and validation set (cross-validation) comprised 500 patients, of whom 270 from Centre 1 with a mean age of 62 ± 14 years for COVID-19 patients, and 57 ± 21 years for negative patients. Out of 270 patients, 149 were males (55%). For centre 2, among the 230 subjects, the mean age was 70 ± 15 years for COVID-19-positive patients, and 70 ± 17 years for negative patients. Out of those 230 patients, 150 were males (65%). The 270 patients who referred to the emergency room of Centre 1 presented fever (87.2%), cough (56.2%) and dyspnoea (40.3%).

### Image analysis by deep learning

Accuracy for the training was 0.99 (95% CI 0.98–1.00). For validation of CXRs (250 of COVID-19 and 250 of non-COVID-19 subjects), our deep learning model was able to automatically classify patients with sensitivity of 0.78 (95% CI 0.74–0.81), specificity of 0.82 (95% CI 0.78–0.85), LR+ of 4.24 (95% CI 3.24–5.55), LR- of 0.27 (95% CI 0.21–0.34), and AUC of 0.89 (95% CI 0.86–0.91) (10-fold cross-validation). For the 110 CXRs of the independent (temporally separated) group of suspect COVID-19 patients, our deep learning system showed sensitivity of 0.80 (95% CI 0.72–0.86), specificity of 0.81 (95% CI 0.73–0.87), LR+ of 4.10 (95% CI 2.09–8.05), LR- of 0.25 (95% CI 0.16–0.41), and AUC of 0.81 (95% CI 0.73–0.87). Table 2 shows a comprehensive list of the performance obtained by the deep learning model.

**Table 2:** Results for the deep learning classifier and human readings of study datasets

COVID-19 positive *versus* negative		
**Classifier validation (cross-validation)**		
	**Positive (*n*)**	**Negative (*n*)**
**Assigned positive**	195	46
**Assigned negative**	55	204
	**Sensitivity**	**Specificity**
	0.78* (0.74–0.81)	0.82* (0.78–0.85)
	**LR+**	**LR-**
	4.24* (3.24–5.55)	0.27* (0.21–0.34)
**Classifier independent testing**		
	**Positive (*n*)**	**Negative (*n*)**
**Assigned positive**	59	7
**Assigned negative**	15	29
	**Sensitivity**	**Specificity**
	0.80* (0.72–0.86)	0.81* (0.73–0.87)
	**LR+**	**LR-**
	4.10* (2.09–8.05)	0.25* (0.16–0.41)
**Human independent testing (Reader 1)**		
	**Positive (*n*)**	**Negative (*n*)**
**Assigned positive**	47	8
**Assigned negative**	27	28
	**Sensitivity**	**Specificity**
	0.64 (0.52–0.74)	0.78 (0.61–0.90)
	**LR+**	**LR-**
	2.86 (1.51–5.39)	0.47 (0.33–0.66)
**Human independent testing (Reader 2)**		
**Assigned positive**	47	5
**Assigned negative**	27	31
	**Sensitivity**	**Specificity**
	0.64 (0.52–0.74)	0.86 (0.71–0.95)
	**LR+**	**LR-**
	4.57 (1.99–10.50)	0.42 (0.31–0.59)

Data are presented as value and 95% confidence interval. *p < 0.005. *COVID-19* Coronavirus disease 2019, *LR+* Positive likelihood ratio, *LR-* Negative likelihood ratio

### Image analysis by radiologists

For the 110 cases from Centre 1 used for the independent testing of the deep learning system, Reader 1 showed sensitivity of 0.64 (95% CI 0.52–0.74), specificity of 0.78 (95% CI 0.61–0.90), LR+ of 2.86 (95% CI 1.51–5.39), and LR- of 0.47 (95% CI 0.33–0.66); Reader 2 showed sensitivity of 0.64 (95% CI 0.52–0.74), specificity of 0.86 (95% CI 0.71–0.95), LR+ of 4.57 (95% CI 1.99–10.50), and LR- of 0.42 (0.31–0.59) (see Table 2).

## Discussion

COVID-19 is a viral infectious disease transmitted through air droplets and close distance contacts caused from infection by SARS-CoV-2. The outbreak of SARS-CoV-2 epidemic has resulted in a global health emergency, more diffuse than the coronavirus severe acute respiratory syndrome (SARS) in 2003, both caused by viruses belonging to the Coronaviridae family [3]. As a matter of fact, on 13 March 2020, the WHO declared the SARS-CoV-2 outbreak a pandemic [19].

Diagnosing the disease quickly and accurately is a clinical need, and CXR is a vital diagnostic tool for COVID-19 in emergency. However, its performance in the diagnosis of COVID-19 cases has not yet been reported by large studies. This study collected a total of 250 COVID-19 patients who had CXR with a positive RT-PCR, enriched with 135 patients with CXR and a negative RT-PCR test, and other 115 non-COVID-19 patients with CXR in an equivalent period preceding the epidemic, to train and test a CNN-based deep learning classifier.

The main finding of our study is that the performance of our deep learning system proved intriguing both at 10-fold cross-validation and when challenged on an independent new dataset.

It is highly likely that a human reading completely informed about history and clinical data or during booming of the epidemic with an increasing prevalence would have been able to strongly increase the sensitivity, but a trade-off could be paid in terms of specificity. This phenomenon is well visible in the case of CT in the recent report by Ai et al. [20] where a 97% sensitivity is counterbalanced by a 25% specificity. The performance of our deep learning system appears interesting for the well balance between the two terms, with 0.80 sensitivity and 0.81 specificity. No a priori selected lung pattern was used to train the deep learning system, in order to avoid human bias or limitations. Such deep learning system includes many convolutional filters that learned a rich feature representation from millions of images of different classes (from low to high level of feature complexity) and used this variety of feature representation for the COVID-19 *versus* non-COVID-19 image classification task.

This constitutes a promising starting point, especially when considering the technical issue regarding bedside CXRs that were evaluated by the deep learning system: only one anteroposterior projection in supine position. This means that there is room for improving CXR performance in these patients. On the one side, the deep learning classifier can be trained on thousands of cases, applying the deep learning general principle: the more data you use for training, a higher performance you get [21]. On the other side, CXR using the standard approach, *i.e*., both the posteroanterior and lateral projections to the patient standing in upright position, could substantially increase the quality of the radiograms and the three-dimensional information provided. However, this “state-of-the-art” approach is not always easy to carry on in the epidemic context, taking into consideration the possible contemporary use. Therefore, while all suspected COVID-19 patients ought to be isolated, this deep learning tool may help guide their clinical workflow, for instance sending patients to thoracic CT when human reading is negative and deep learning classifier reading is positive.

It is important to recognise that the role of CXR in patients’ evaluation depends on the severity of infection in the individual patient, as well as on the COVID-19 prevalence in the community. In individuals who are asymptomatic or have mild disease, the sensitivity of CXR could fail if performed in the first 48 h from the onset of symptoms. Individuals with very mild disease may eventually have positive RT-PCR results but would have been missed by early CXR. Conversely, CXR should be most useful in patients who are acutely ill and symptomatic in areas with relatively high prevalence, such as Lombardy, Italy in spring 2020. In this scenario, patients with the clinical condition and CXR findings attributable to COVID-19 could be considered as possibly infected by the virus when the first RT-PCR test result is still not available or negative.

Since the beginning of the pandemic, there have been numerous published studies that use machine learning or CNNs for diagnosing COVID-19 from CXR. Among these, the majority [10–14] used transfer-learning techniques for automatically classifying COVID-19, based on different pretrained CNNs (*e.g*., VGG-19, SqueezeNet, DenseNet); in some of them, some optimisations were also performed (*e.g*., Bayesian optimisation by Ucar et al. [13] or hierarchical classification by Pereira et al. [14]).

As a first point of comparison, none of the considered published papers [10–14] used an independent testing set (neither temporally nor spatially independent) to obtain an unbiased evaluation of the performance of their machine learning classifiers, which was made instead in the present paper. Thus, the performance obtained by the referenced literature may suffer from overfitting issues.

Furthermore, the referenced works [10–14] did not perform a comparison between the performance obtained by the machine learning classifiers and those obtained by expert radiologists. Our study compares the performance of a deep learning classifier to the radiologists’ reading for COVID-19 diagnosis, thus providing interesting information about the potential adoption of the proposed classifier as a second reader to support decision in clinical practice.

As a last point of comparison, such works [10–14] used publicly available anonymised image sets for normal or non-COVID-19 CXRs collected by a group of imaging centres before the COVID-19 pandemic. As COVID-19 CXRs, instead, these studies used publicly available anonymised image sets collected by a different group of imaging centres during the COVID-19 pandemic.

Thus, in these published papers, the intrinsic systematic image differences among image sets of normal or non-COVID-19 CXRs distinct from image sets of COVID-19 CXRs (*e.g*., distinct acquisition protocols, imaging systems, subjects origin) may have inflated the final classification performance of the deep learning models.

For example, most of the published papers used non-COVID-19 CXRs from the well-known Kaggle database “Chest X-Ray Images (Pneumonia)” [22]. However, this database is composed of CXRs of normal subjects and patients with non-COVID19 pneumonia (other community-acquired pneumonia) obtained from retrospective cohorts of paediatric patients of 1 to 5 years old (from Guangzhou Women and Children’s Medical Center, Guangzhou). If these CXRs are classified against nonpaediatric COVID-19 patients, this may heavily affect the classification performance of the deep learning models.

**Fig. 3:**
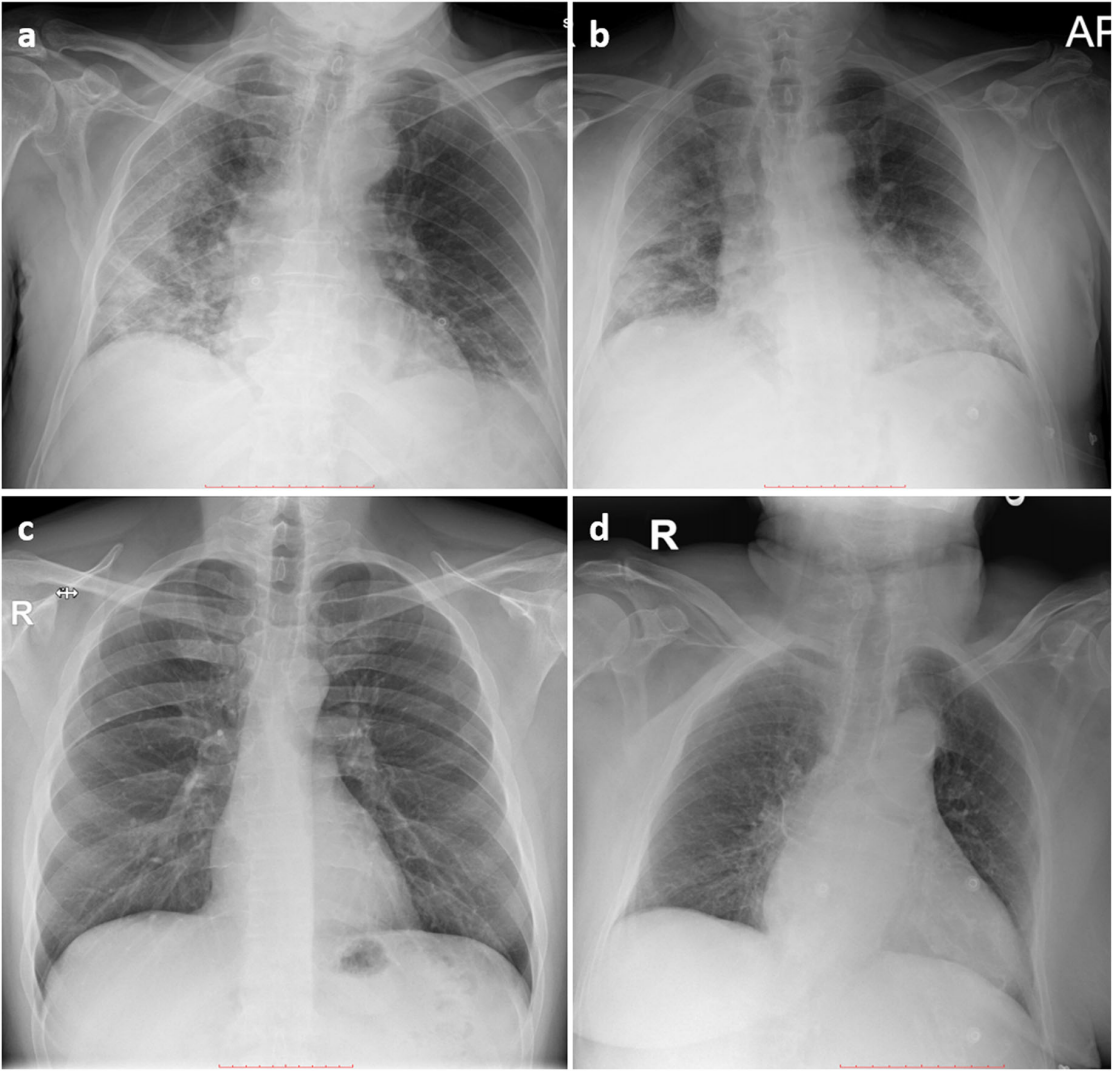
Sample chest x-ray examinations from patients included in the study sample. Posteroanterior projection in a COVID-19-positive 75-year-old male patient (**a**), bedside anteroposterior projection in a COVID-19-positive male patient (**b**), posteroanterior projection in a negative 42-year-old male patient (**c**) and bedside anteroposterior projection in a negative 88-year-old female patient (**d**)

This study has some limitations. First, we trained our model on a limited number of cases, from the same geographical area. We could improve performances and generalisability of our model by adding new images, in particular from different geographical regions than Lombardy. Second, the independent testing set was only temporally separate but not geographically separate from the training one and also relatively small. This may lead to an algorithm well-fitted on a local scale, with an unknown performance on distant cohorts. In this regard, future studies should be focused on testing the algorithm on CXR image sets originating from other populations and geographical areas, and eventually reducing overfitting by including such datasets in training. For a worldwide generalisation, the algorithm should probably be retrained and tuned also including CXR images from noncaucasian races such as Asian and African ones. Third, we did not include other data such as clinical conditions such as symptoms and pulse oximeter data as complementary information to be given to the deep learning model and the human readers, a perspective to be explored in future studies. Moreover, the dataset used to train the algorithm was designed to give a binary decision (COVID-19 *versus* non-COVID-19). However, this decision may be dependent on disease severity. The dataset was enriched with x-ray chest radiographs of non-consecutive sex- and age-matched patients but it was not matched for the severity of lung abnormalities; thus, the algorithm is not currently able to classify the severity of lung abnormalities but only the presence or absence of lung abnormalities associated to COVID-19-positive patients (Fig. 3). A further limitation of the algorithm may be posed by the inclusion of both anteroposterior (bedside) and posteroanterior (standard) CXR projections in the training dataset, whereas only bedside, anteroposterior CXRs were present in the independent testing dataset. Indeed, concerning patient position during CXR exams, as reported in the “Methods” section, while patients from Centre 1 only had bedside anteroposterior (AP) CXR exams, patients from Centre 2 had either AP or PA projections, the latter belonging mostly to healthy controls or to healthier patients. Thus, the deep learning system was trained on a dataset composed by frontal PA or AP images. While this could have led to a source of bias, with AP projections being linked to patients with more severe disease, and therefore COVID-19 cases, and PA images being linked to healthy subjects, the good LR- seems to suggest that it was not the case, as the algorithm was able to correctly identify a substantial number of cases of the independent testing set as negative. As a matter of fact, the performance of the algorithm seemed to err towards false negative interpretation, as opposed to vice versa, further suggesting that the presence of different projections did not hinder the performance on the testing set.

In conclusion, we preliminarily showed that a CNN-based deep learning system applied to bedside CXR in patients suspected to be positive COVID-19, even though trained on a limited number of cases, allowed to reach a 0.80 sensitivity and a 0.81 specificity in an independent temporally separate patient group. The system could be used as a second opinion tool in studies aimed at assessing its usefulness for improving the final sensitivity and specificity in different geographical and temporal setting. Its performance could be improved by training on larger multi-institutional and multi-geographical datasets, and the role of the algorithm as the second reader of CXR images could be assessed in different instances in patients suspected for SARS-CoV-2 infection, especially as several countries are facing repeated waves of the COVID-19 pandemic. This deep learning tool may help guide the clinical workflow, for instance sending patients to thoracic CT when human reading is negative and results from the deep learning classifier are positive.

## Data Availability

The datasets used and/or analysed during the current study are available from the corresponding author on reasonable request.

## Abbreviations

AUC: Area under the curve
CI: Confidence interval
CNN: Convolutional neural network
COVID-19: Coronavirus disease 2019
CT: Computed tomography
CXR: Chest x-ray
LR-: Negative likelihood ratio
LR+: Positive likelihood ratio
PCR: Polymerase chain reaction
RT-PCR: Reverse transcriptase polymerase chain reaction
SARS-CoV-2: Severe acute respiratory syndrome coronavirus 2
